# Deciphering the Transcriptional Response Mediated by the Redox-Sensing System HbpS-SenS-SenR from Streptomycetes

**DOI:** 10.1371/journal.pone.0159873

**Published:** 2016-08-19

**Authors:** Tobias Busche, Anika Winkler, Ina Wedderhoff, Christian Rückert, Jörn Kalinowski, Darío Ortiz de Orué Lucana

**Affiliations:** 1 Microbial Genomics and Biotechnology, Center for Biotechnology, Bielefeld University, Universitätsstraße 27, 33615, Bielefeld, Germany; 2 Applied Genetics of Microorganisms, Department of Biology and Chemistry, University of Osnabrueck, Osnabrueck, Barbarastraße 13, 49076, Osnabrueck, Germany; Centre National de la Recherche Scientifique, Aix-Marseille Université, FRANCE

## Abstract

The secreted protein HbpS, the membrane-embedded sensor kinase SenS and the cytoplasmic response regulator SenR from streptomycetes have been shown to form a novel type of signaling pathway. Based on structural biology as well as different biochemical and biophysical approaches, redox stress-based post-translational modifications in the three proteins were shown to modulate the activity of this signaling pathway. In this study, we show that the homologous system, named here HbpSc-SenSc-SenRc, from the model species *Streptomyces coelicolor* A3(2) provides this bacterium with an efficient defense mechanism under conditions of oxidative stress. Comparative analyses of the transcriptomes of the *Streptomyces coelicolor* A3(2) wild-type and the generated *hbpSc-senSc-senRc* mutant under native and oxidative-stressing conditions allowed to identify differentially expressed genes, whose products may enhance the anti-oxidative defense of the bacterium. Amongst others, the results show an up-regulated transcription of genes for biosynthesis of cysteine and vitamin B_12_, transport of methionine and vitamin B_12_, and DNA synthesis and repair. Simultaneously, transcription of genes for degradation of an anti-oxidant compound is down-regulated in a HbpSc-SenSc-SenRc-dependent manner. It appears that HbpSc-SenSc-SenRc controls the non-enzymatic response of *Streptomyces coelicolor* A3(2) to counteract the hazardous effects of oxidative stress. Binding of the response regulator SenRc to regulatory regions of some of the studied genes indicates that the regulation is direct. The results additionally suggest that HbpSc-SenSc-SenRc may act in concert with other regulatory modules such as a transcriptional regulator, a two-component system and the *Streptomyces* B_12_ riboswitch. The transcriptomics data, together with our previous *in vitro* results, enable a profound characterization of the HbpS-SenS-SenR system from streptomycetes. Since homologues to HbpS-SenS-SenR are widespread in different actinobacteria with ecological and medical relevance, the data presented here will serve as a basis to elucidate the biological role of these homologues.

## Introduction

Streptomycetes are Gram-positive soil-dwelling bacteria with a complex developmental life cycle that includes formation of aerial mycelia and spores [1]. They synthesize a wide repertoire of chemically distinct low-molecular-weight compounds including medically relevant antibiotics, anti-tumors agents and immunosuppressants [2]. Streptomycetes have a special role in soil ecology since they secrete many hydrolytic enzymes which help during the initial breakdown of insoluble organic material such as crystalline cellulose, xylan and chitin [3, 4]. The secretion of secondary metabolites, enzymes and enzyme inhibitors is closely associated with the ability of streptomycetes to interact with other bacteria, fungi, plants and insects within various ecological niches [2, 3].

The coordination of the complex developmental life cycle, synthesis of secondary metabolites, interaction with other organisms, as well as the response to highly variable environmental conditions requires the presence of different signal processing pathways. Streptomycetes have large genomes, usually between 8.7 Mb and 11.9 Mb [5, 6]. It is worth noting that 12.3% of the total open reading frames (ORF) within the genome of *Streptomyces coelicolor* A3(2), the model organism among the genus *Streptomyces*, encode proteins with predicted regulatory functions including transcription factors, sigma factors and two-component systems (TCS) [7]. In comparison to other bacteria, streptomycetes possess a high number of TCS [8], but only few have been investigated experimentally. In general, the prototypical TCS consists of a sensor, which is a membrane-embedded histidine kinase (SK), and a cytosolic response regulator (RR), which, depending on its phosphorylation state, interacts with promoter regions to regulate DNA transcription [9, 10].

A standard SK contains a periplasmic input sensing domain that detects environmental stimuli, and a cytosolic autokinase domain linked by a transmembrane region. Sequence homology among sensing domains from different SKs is typically low, reflecting the diverse nature of signals detected. Topology predictions showed that PAS (PER-ARNT-SIM) domains are the most frequent sensor domains found in SKs [10]. They were shown to play a role during protein-protein interactions and ligand binding [11]. Structural studies of the PAS domain from the SK CitA showed that the binding of citrate, the signal molecule, causes a conformational change within the PAS domain that, in turn, leads to activation of the phosphorylation cascade [12].

Recent studies have shown that some TCSs require accessory proteins for signal sensing [13, 14]. One of the best biochemically and structurally studied systems is the three-component system HbpS-SenS-SenR from *Streptomyces reticuli* (*S*. *reticuli*). HbpS is a secreted octameric protein that binds iron ions as well as the tetrapyrroles heme and aquo-cobalamin (vitamin B_12a_) [15–17]. It is also interesting to note that the crystal structure of HbpS is similar to the so-called GAF (cGMP-specific phosphodiesterases, Adenylyl cyclases and FhlA) domains which have a PAS-fold and are located, for example, within the sensing domains of DosS and DosT from *Mycobacterium tuberculosis* [18–20]. Both are two-component SKs that use heme for sensing. HbpS specifically interacts with the sensing domain of the SK SenS and modulates its autophosphorylation [21].

Based on the crystal structure of HbpS and fluorescence resonance energy transfer (FRET), circular dichroism (CD) and electron paramagnetic resonance (EPR) spectroscopic studies, we showed that the presence of reactive oxygen species (ROS) causes oxidative modifications (i.e. dityrosine formation and carbonylation) accompanied by overall conformational changes within the HbpS octamer [15, 22, 23]. These induce autophosphorylation of the SK SenS that in turn phosphorylates the RR SenR which activates the transcription of the *cpeB* gene encoding the mycelium-associated catalase-peroxidase CpeB. This enzyme provides *S*. *reticuli* with a non-stressed environment that protects freshly secreted HbpS from oxidation [23]. This HbpS form inhibits SenS autophosphorylation, leading to down-regulation of the HbpS-SenS-SenR signalling cascade [14].

In addition to catalases and peroxidases, bacterial cells contain other scavenging enzymes including superoxide dismutases (SODs) or alkylhydroperoxide reductases (AHPs) that counteract the hazardous effects of ROS (i.e. hydrogen peroxide, H_2_O_2_; hydroxyl radical, OH^•^; superoxide anion, O_2_^-^). SODs maintain the concentration of O_2_^-^ in low limits through its dismutation to H_2_O_2_ and O_2_. Catalases promote the breakdown of H_2_O_2_, leading to O_2_ and H_2_O. AHPs are two-component thiol-based peroxidases which reduce H_2_O_2_ to H_2_O through the transfer of electrons from NADH to the peroxide [24]. In addition to these enzymes, cells produce low-molecular-weight thiols (i.e. glutathione, cysteine, mycothiol or coenzyme A) that play a key role in the anti-oxidative stress response by maintaining an intracellular reducing environment [25]. While glutathione is utilized as a redox buffer in Gram-negative bacteria, mycothiol is the major thiol in actinomycetes (e.g. *Streptomyces*, *Mycobacteria*, and *Corynebacteria*) [26, 27]. An additional mechanism of defense against oxidative stress is the protein-mediated sequestration of high quantities of ferrous ions that might be involved in the generation of hydroxyl radicals via the Fenton reaction [28]. This mechanism has been described for ferritins and Dps proteins [29, 30]. Similarly, the HbpS protein from *S*. *reticuli* binds high quantities of ferrous ions and oxidizes them to the ferric form, making them inaccessible for the Fenton reaction [16].

**Fig 1:**
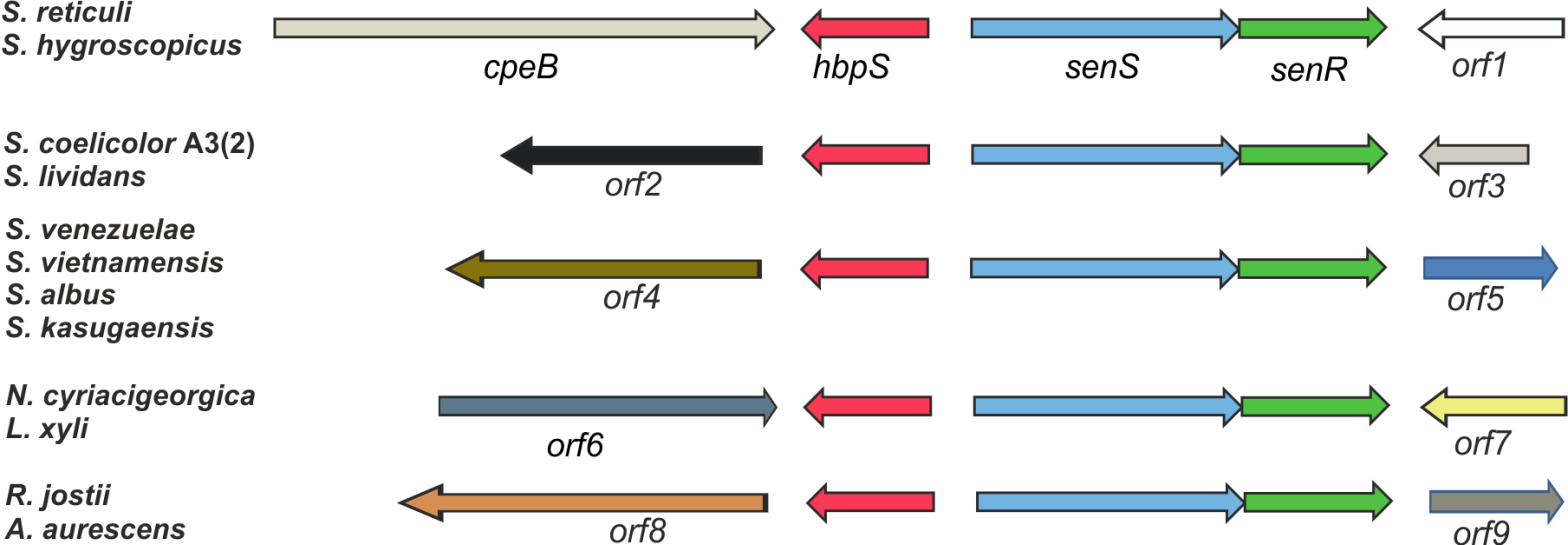
Relative location and transcriptional orientation of *hbpS*, *senS*, *senR* and related genes on different actinobacterial genomes. The catalase-peroxidase gene (*cpeB*) and *orf1* are located upstream and downstrean, respectively, of the *hbpS*-*senS*-*senR* gene cluster on the genome of *S*. *reticuli* and *S*. *hygroscopicus*. In the other indicated *Streptomyces* strains as well as in *Nocardia cyriacigeorgica* (*N*. *cyriacigeorgica*), *Leifsonia xyli* (*L*. *xyli*), *Rhodococcus jostii* (*R*. *jostii*) and *Arthrobacter aurescens* (*A*. *aurescens*), a *cpeB*-like gene is absent. *orf*1-9 encode proteins with different predicted functions. *hbpS* and *hbpS*-like genes are marked in red, *senS* and *senS*-like in light-blue, and *senR* and *senR*-like in green.

Sequence comparisons allowed the identification of HbpS-like proteins within Gram-positive and Gram-negative bacteria. Remarkably, only the *hbpS*-like genes from a number of actinobacteria (including different *Streptomyces sp*., *Arthrobacter aurescens*, *Rhodococcus jostii* RH1, *Nocardia cyriacigeorgica* GUH-2 and *Leifsonia xyli*) are clustered with the *hbpS*, *senS* and *senR* genes in the same relative transcriptional orientation [31] (Fig 1). The *S*. *reticuli hbpS*-*senS*-*senR* genes are additionally clustered with *cpeB*, encoding the the catalase-peroxidase CpeB. This gene is, however, absent in the vicinity of most of the *hbpS*-*senS*-*senR* homologous genes (Fig 1).

The aim of the present work is to gain deeper insight into the anti-oxidative stress response mediated by the HbpS-SenS-SenR system *in vivo*. For that, we used the model streptomycete, *Streptomyces coelicolor* A3(2). We first generated a *hbpSc-senSc-senRc* disruption mutant and the respective complemented mutant, and comparative growth assays were performed. Total RNA was isolated and analyzed by RNA-sequencing (RNA-Seq). Comparative analyses allowed the identification of differentially expressed genes, which are under the control of HbpSc-SenSc-SenRc. These results were validated by qRT-PCR and DNA-binding studies using the isolated regulator protein SenRc.

## Materials and Methods

### Strains, media and culture conditions, and plasmids

*Streptomyces coelicolor* A3(2) (*S*. *coelicolor*) wild-type as well as the mutants ∆*hsr* and ∆*hsr* + HSR (see below) were cultivated in complete (R2) liquid medium [32]. *Streptomyces* spores were obtained as previously described [16]. *Eschericchia coli* (*E*. *coli*) strains BL21(DE3)pLysS and DH5α were cultivated in LB medium. The pBR322 derivative containing the hygromycin resistance cassette (p45Ωhyg) [33], pUC18 [34], pGM160, a bifunctional temperature-sensitive *Streptomyces* vector [35], and the expression plasmid vector pETM11 [36] were used.

### Chemicals and enzymes

Chemicals for SDS- and native-PAGE were obtained from ROTH. Hydrogen peroxide (H_2_O_2_), sybr green, diamide and oligonucleotides were purchased from Sigma Aldrich. Molecular weight markers for DNA and protein, restriction enzymes, T4 Ligase, and DNA polymerase for PCR were obtained from Thermo Scientific or New England BioLabs.

### Isolation of DNA, transformation and hybridization experiments

Chromosomal DNA of *Streptomyces* strains was isolated after growth in a sucrose-containing R2 medium for 2 days [32]. Isolation of plasmids and extraction of DNA from gels were performed using the Qiagen mini and midi plasmid preparation, and the gel extraction kits. DNA was cleaved with various restriction enzymes according to the suppliers’ (New Englad BioLabs; Thermo Scientific) instructions. Ligation was performed with T4 ligase. Gel electrophoresis was carried out in 0.7–2% agarose gels using TBE buffer. Plasmids were used to transform *E*. *coli* DH5α by electroporation or *E*. *coli* BL21(DE3)pLysS with the CaCl_2_ method [37]. Isolation of *S*. *coelicolor* protoplasts and their transformation with plasmids were done as described [32]. DNA fragments of the restricted chromosome from *Streptomyces* strains were transferred onto nylon membranes as described [37]. The hybridization probes were labelled using Klenow enzyme and digoxigenin-11-dUTP (Roche). Hybridization and immunological detection were carried according to the standard procedures [37].

### Generation of the *S*. *coelicolor hbpSc*-*senSc*-*senRc* disruption mutant (∆*hsr*)

The DraI fragment of p45Ωhyg containing the hygromycin-resistant cassette (*hyg*) was ligated with the longer HincII fragment of pUC18. The resulting plasmid was named pUCHyg. The region (~1000 bp) downstream of *hbpSc* (SCO4274) was amplified from the *S*. *coelicolor* chromosome using the primers LHinfor and LPstrev (S1 Table). The HindIII/PstI-cleaved PCR product was ligated with the longer HindIII-PstI fragment of pUCHyg, and the ligation mixture was used to transform *E*. *coli* DH5α. Hygromycin-resistant *E*. *coli* transformants were selected, and the correctness of the resulting plasmid construct pULHyg was confirmed by sequencing. The region (~1000 bp) downstrean of *senRc* (SCO4276) was amplified from the *S*. *coelicolor* chromosone using the primers RBamfor and RKpnrev (S1 Table), and subsequently cleaved with BamHI and KpnI. The restriction product was ligated with the longer BamHI-KpnI fragment of pUCLHyg. After transformation, the resulting plasmids were isolated from *E*. *coli* DH5α transformants. The resulting construct pUCLHygR, containing the hygromycin resistance cassette flanked by the downstream region of both *hbpSc* and *senRc*, was used for further cloning experiments. To simplify the next clonig step, we cleaved the bifunctional and temperature-sensitive plasmid pGM160 carrying the thiostrepton resistance (*tsr)* gene with HindIII, extracted the small HindIII-HindIII fragment, and ligated the longer one. The resulting plasmid was named pGM160∆H. This plasmid was cleaved with BamHI and pUCLHygR with *Kpn*I. Both linearized plasmids were subsequently blunt-ended using the Klenow enzyme and cleaved with HindIII. The DNA fragment from pUCLHygR containining *hyg* and the flanking regions was ligated with the HindIII-blunt end fragment from pGM160∆H. After transformation in *E*. *coli* DH5α, the resulting plasmid construct, named pGMLHygR, was isolated and analyzed using restriction enzymes. pGMLHygR was used to transform 50 μl of protoplasts (~10 ^9^/ml) of *S*. *coelicolor*. After their regeneration, selection occurred at 30°C for resistance against thiostrepton (25 μg/ml). To prevent autonomous replication of pGMLHygR, thiostrepton-resistant colonies were streaked on complete medium containing hygromycin (50 μg/ml) and incubated at 37°C until sporulation occurred. Hygromycin-resistant spores were restreaked several times and incubated at 37°C. To characterize the disruptions, chromosomal DNAs of hygromycin-resistant and thiostrepton-sensitive *S*. *coelicolor* strains were isolated and cleaved with restriction enzymes. DNAs were subjected to Southern analysis with probes for the antibiotic resistance gene and those genes that were expected to be disrupted.

### Cloning of *hbpSc*-*senSc*-*senRc* for complementation analyses

The overall strategy consisted of cloning the DNA region comprising the *hbpSc-senSc-senRc* gene cluster in to the shuttle vector pWHM3 that can replicate in *Streptomyces*. It is worth mentioning that the *hbpSc-senSc-senRc* cluster contains its own regulatory elements located in the intergenic region between *hbpSc* and *senSc*. To amplify *hbpSc*-*senSc*-*senRc* from the *S*. *coelicolor* chromosome two PCR reactions were necessary. With the first one the DNA region containing *hbpSc* and part of *senSc* was amplified using the primers PKpnFor and PBamRev (S1 Table). The PCR product was digested with KpnI and BamHI, ligated with KpnI/BamHI-cleaved pUC18 and subsequently transformed into *E*. *coli* DH5α. Plasmids of selected ampicillin-resistant transformants were checked by sequencing. The plasmid construct carrying the desired DNA fragment was named pUHS. With the second PCR reaction the remaining part of *senSc* plus *senRc* was amplified using the primers PBamFor and PHinRev (S1 Table). The PCR product was digested with BamHI and HindIII, ligated with BamHI/HindIII-cleaved pUHS and subsequently transformed into *E*. *coli* DH5α. Plasmids of selected transformants were checked by sequencing. The plasmid construct carrying the desired DNA fragment was named pUHSR. The EcoRI-HindIII fragment of pUHSR containing *hbpSc*-*senSc*-*senRc* was ligated with the EcoRI/HindIII-cleaved cloning vector pWHM3 and transformed into *E*. *coli* DH5α. The correctness of the cloning was checked by restriction. The plasmid construct obtained was named pWHSR and used to transform protoplasts of *S*. *coelicolor* ∆*hsr*. Thiostrepton-resistant transformants were named *S*. *coelicolor* ∆*hsr* pWHSR, abbreviated as ∆*hsr* + HSR.

### Isolation of total RNA for RNA-Seq

To obtain well-grown mycelia, spores (10^9^) of *S*. *coelicolor* (WT and ∆*hsr* mutant) were inoculated in 10 ml R2 medium and grown as standing culture at 30°C for 16 h and afterwards on a rotary shaker for 16 h after the addition of 90 ml R2 medium. The cultures were washed four times in minimal medium without supplement. The mycelia were suspended in 50 ml R2 medium and divided in two 25 ml-portions, one of which contained H_2_O_2_ (0.15 mM). Cultivation was continued at 30°C on a rotary shaker for two hours. Mycelia were harvested by centrifugation and the mycelia pellets were stored at -80°C. Total RNA was isolated from two biological replicates using in part a previously reported protocol Ortiz de Orue Lucana and Schrempf [38] and the RNeasy Mini Kit along with a DNase Kit (both from Qiagen). Briefly, mycelia were washed with 25 mM Tris, pH 7.5. 1 g mycelia was resuspended in a 3 ml-solution of 4 M guanidine thiocyanate, 25 mM sodium citrate, pH 7, 0.5% laurylsarcosine and 0.1 M 2-mercaptoethanol, and treated with 1 vol. glass beads by vortexing for 3 min. The glass beads and the cell debris were removed by centrifugation. 500 μl of the supernatant were used for isolation according to the protocols described in the RNeasy Mini and DNase Kit. The quality of the RNA was controlled on a 2% agarose gel, and it was tested for possible DNA contaminations by PCR.

### Transcriptome library preparation and sequencing

RNA concentrations and quality were determined using Trinean Xpose (Gentbrugge, Belgium) and Agilent RNA Nano 6000 kit on Agilent 2100 Bioanalyzer (Agilent Technologies, Böblingen, Germany). Ribo-Zero rRNA Removal Kit (Bacteria) from Illumina (San Diego, CA, USA) was used to deplete ribosomal RNA. The quality of rRNA-depleted RNA samples was checked on Agilent 2100 Bioanalyzer. cDNA libraries were constructed using the TruSeq Stranded mRNA Library Prep Kit (Illumina,San Diego, CA, USA), and subsequently sequenced paired-end on an Illumina MiSeq system (San Diego, CA, USA) using 75 bp read length. RNA-Seq data were deposited in the ArrayExpress database (http://www.ebi.ac.uk/arrayexpress/) under the accession number E-MTAB-4833.

### Transcriptome assembly

Trimmed reads (26 nt) were mapped to the *S*. *coelicolor* genome sequence (accession number NC_003888) [7] with SARUMAN [39], allowing for up to one error per read. The forward and reverse read, if both present and with a maximum distance of 1 kb, were combined to one read that contains the reference sequence as insert. Paired mappings with a distance > 1 kb were discarded, and paired reads with either only the forward or only the reverse read mapping were retained as single mapping reads, as described previously [40]. ReadXplorer 2.1.0 [41] was used for visualization of short read alignments.

### Differentially expressed genes

Reads per kilobase per million mapped reads (RPKM) [42] were calculated based on the raw read counts per CDS plus one pseudo read. For differential RNA-Seq analyses the signal intensity value (A-value) was calculated by average (log2) RPKM of each gene and the signal intensity ratio (M-value) by the difference of (log2) TPM. The evaluation of the differential RNA-Seq data was done using a RPKM cut-off of ≥ 30, an A-value cut-off of > 2.0, and an individual M-value cut-off for each differential gene expression analysis. The latter was chosen based on the minimal M-value cut-off determined by applying a significance level of 1% to the experiment with the assumption that the majority of genes is not differentially transcribed. Thus, 99% of all M-values should fall within this range. Therefore, the standard deviation (STDEV) for all M-values was calculated and the minimal M-value cut-off was set to m = 2.58 * STDEV. Genes with M-values of higher/equal than +1.5 or lower/equal than −1.5 (corresponding to fold changes of 2.8 and 0.4, respectively) were considered to be differentially transcribed.

### Quantitative real time PCR (qRT-PCR) experiments

Total RNA of *S*. *coelicolor* (WT and ∆*hsr*) was isolated from three biological replicates that grew in the absence or presence of 0.15 mM H_2_O_2_ for 2 h. 120 ng of total RNA per 20 μl reaction were employed for qRT-PCR using the SenSciFAST SYBR Hi-ROX One-Step Kit (Bioline, Luckenwalde, Germany) on a LightCycler 96 instrument (Roche Diagnostics, Risch-Rotkreuz, Switzerland). Primers (S1 Table) were designed to amplify approximately 100 bp intragenic fragments. Melting curve analysis was performed to exclude the formation of side products or primer dimers. Crossing points (CP) were determined as second derivative maximums from the obtained fluorescence curves with the LightCycler software (Roche Diagnostics). The differences in gene expression were determined by comparing the CPs of three samples measured in duplicate. The calculation of the average CP was performed by first calculating the averages for each set of technical replicates and then by calculating the average of the three biological replicates. For each set of three biological replicates, the standard deviation was calculated (assuming a normal distribution of the CPs) and the combined standard deviation for the DeltaCP was approximated using the standard calculation for the propagation of uncertainty (assuming non-correlated errors).

### Cloning, expression and isolation of the regulator SenRc

The *senRc*-coding region was amplified by PCR from the *S*. *coelicolor* chromosome using the primers PRForNco (S1 Table) consisting of an NcoI restriction site, followed by the sequence encoding the N-terminal amino acids of SenRc, and PRRevHind (S1 Table) coding for the C-terminal amino acids of SenRc, followed by the HindIII restriction site. The PCR-product was digested with NcoI and HindIII, ligated with NcoI/HindIII-cleaved pETM11 [36] and subsequently transformed into *E*. *coli* DH5α. Sequencing of the resulting plasmid (pETSenRc), confirmed the presence of the *senRc* gene in frame fusion with the His_6_-tag codons. pETSenRc was used to transform *E*. *coli* BL21(DE3)pLysS. Protein expression was induced at OD_600_ = 0.6 at 37°C with 1 mM IPTG (isopropyl-ß-D-thiogalactopyranoside) for 4 h. Cell pellets were resuspended and washed with a chilled solution W (100 mM Tris-HCl, pH 8, 150 mM NaCl) containing DNaseI (1 μg/ml), and then lysed by ultrasonication (Branson sonifier, 5 x 10 s, with 10 s-intervals). Cell debris were centrifuged at 30 000 g at 4°C.

The supernatant containing soluble proteins was incubated with Ni^2+^-NTA agarose beads in the presence of an additional 25 mM imidazole. The resin was washed with solution W supplemented with 30 mM imidazole, and then the protein was eluted with solution W containing 250 mM imidazole. To separate imidazole from the protein solution, this was dialysed against solution W containing 2 mM EDTA and 2 mM DTT, and subsequently against solution W alone. The Ni^2+^-NTA affinity chromatography and dialysis were repeated. The homogeneity of the His-tag-SenRc protein was analysed by SDS-PAGE. Protein concentration was calculated using the Bradford method [43].

### Electrophoretic mobility shift assays (EMSA)

The upstream regions (~200 bp) of selected genes were amplified with corresponding primers (S1 Table) from the chromosome of *S*. *coelicolor*. 25 ng of the desired DNA fragment was incubated in DNA-binding buffer (10 mM Tris-HCl, pH 7.5, 5% glycerol, 40 mM KCl, 1 mM MgCl_2_, 2 mM DTT and 10 μg/ml BSA) with increasing amounts of His-tag-SenRc at 30°C for 15 min. The final volume of the reaction mixture was 30 μl. Band shifts were analysed by subsequent electrophoresis on a 7% polyacrylamide gel. Gels were run at 60 V for 2 h and products were visualized by DNA-staining using sybr green. EMSAs were repeated three times.

To generate mutated DNA-binding sites of SenRc, the two-step PCR technique was used. In the first step, chromosomal DNA of *S*. *coelicolor* was used as template for PCR. The reactions additionally contained flanking primers 6102for and 6102rev as well as overlapping primers 6102*for and 6102*rev (S1 Table). In the first step 6102for was combined with 6102*rev, whereas 6102*for with 6102rev. In the second step, the obtained PCR products (as template) and the flanking primers were used. The same procedure was followed to mutate the predicted DNA-binding site in front of SCO4229 using the indicated primers in S1 Table.

### Growth assays

R2 agar plates lacking stressors or containing either 0.15 mM H_2_O_2_ or 0.6 mM diamide were used. One μl of either 10^9^/ml (undiluted) or diluted spores (1:2, 1:5, 1:10, and 1:25) from the studied *S*. *coelicolor* strains (WT, ∆*hsr*, ∆*hsr* + HSR) was dropped onto the agar plates that were subsequently incubated at 30°C for three days.

## Results

### The *S*. *coelicolor* HbpSc-SenSc-SenRc system is involved in anti-oxidative stress response

To characterize the transcriptional response mediated by the HbpSc-SenSc-SenRc system in *S*. *coelicolor*, we firstly disrupted the *hbpSc-senSc-senRc* gene cluster by insertional inactivation. For that, the temperature-sensitive plasmid pGMLHygR carrying a thiostrepton resistance gene was constructed (see Materials and Methods). It contains additionally the hygromycin-resistance cassette flanked by cloned chromosomal DNA regions downstrean of *hbpSc* and *senRc*, respectively. *S*. *coelicolor* wild-type (WT) was transformed with pGMLHygR and hygromycin-resistant and thiostrepton-sensitive transformants were selected. Southern blot and RNA-Seq analysis (not shown) confirmed the disruption of *hbpSc-senSc-senRc*. The disruption mutant *S*. *coelicolor* ∆*hbpSc-senSc-senRc* was named ∆*hsr*. This mutant was subsequently complemented with the plasmid pWHSR containing *hbpSc-senSc-senRc* (see Materials and Methods). The resulting complemented mutant was named ∆*hsr* + HSR.

**Fig 2:**
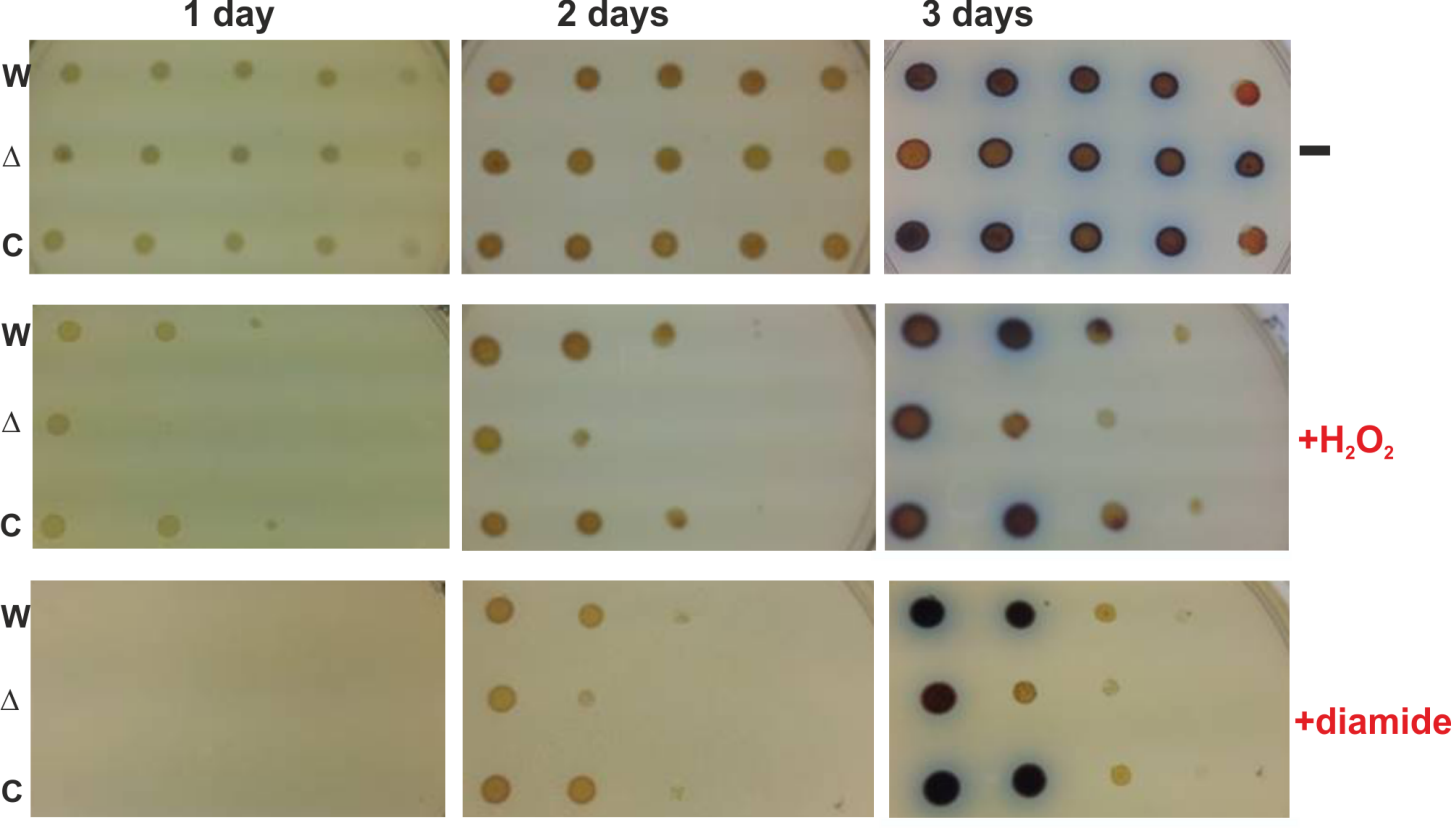
Growth of *S*. *coelicolor* strains under different oxidative-stressing conditions. 10^6^, 5x10^5^, 2x10^5^, 10^5^, 4x10^4^ (from left to right) spores of *S*. *coelicolor* wild-type (W), *S*. *coelicolor ∆hsr* (∆) and *S*. *coelicolor ∆hsr* + HSR (C) were dropped onto agar plates lacking (-) or containing either 0.15 mM hydrogen peroxide (+H_2_O_2_) or 0.6 mM diamide (+diamide). Plates were subsequently incubated at 30°C for three days. The showed plates are representative for three independent growth assays with the same outcome.

Our previous studies showed that disruption of either *hbpS* or *senS-senR* in *S*. *reticuli* led to an increase of sensitivity of this bacterium against different stressors including the redox-cycling compound plumbagin, H_2_O_2_, the thiol-specific oxidant diamide or high concentrations of iron ions [44, 45]. In this work, we compared the growth of *S*. *coelicolor* WT, ∆*hsr* and ∆*hsr* + HSR by plate assays. Spores of the strains were spotted on R2-agar plates lacking or containing either H_2_O_2_ (0.15 mM) or diamide (0.6 mM), and the growth was followed at 30°C over 3 days. The plate assays showed that ∆*hsr* is more sensitive to both stressors than the WT and ∆*hsr* + HSR (Fig 2), indicating that *hbpSc-senSc-senRc* provides *S*. *coelicolor* with a protection mechanism on conditions of oxidative stress.

### RNA-Seq reveals genes that are under the control of HbpSc-SenSc-SenRc

Four TruSeq stranded mRNA libraries were prepared from total RNA that was extracted from two biological replicates of both *S*. *coelicolor* WT and ∆*hsr* mutant cultivated in medium lacking or containing H_2_O_2_ (0.15 mM) for 2 h. The libraries were subsequently sequenced paired end with a read length of 75 nt. 6 to 10.3 million reads were generated from the individual libraries. After mapping of reads to the *S*. *coelicolor* genome, forward and reverse reads (if both present and with a maximum distance of 1 kb) were combined into one read that contained the reference sequence as inserted. Paired mappings with a distance > 1 kb were discarded, and paired reads with either only the forward or only the reverse read mapping were retained as single mapping reads (S2 Table).

**Fig 3:**
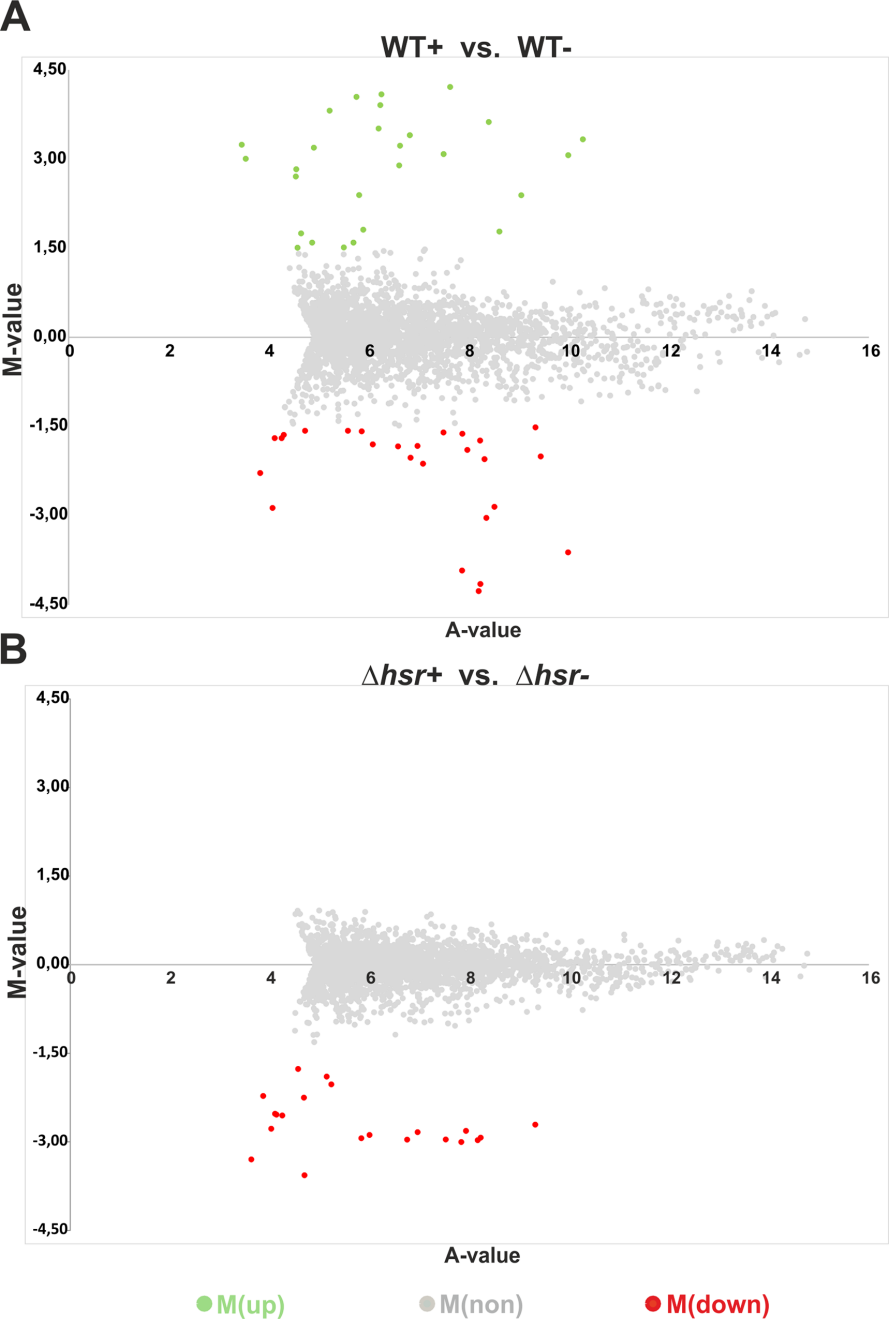
Identification of differential expression. RNA-Seq datasets of stressed (WT+ or *∆hsr*+) and non-stressed (WT- or *∆hsr*-) *S*. *coelicolor* wild-type (A) and *S*. *coelicolor ∆hsr* (B) were used to generate plots, which show up-regulated [M > 1.5, green circles, M(up)], down-regulated [M < -1.5, red circles, M(down)] and non-regulated [M between +1.5 and -1.5, grey circles, M(non)] genes. M-values (Y-axis) and A-values (X-axis) were calculated as indicated in the Material and Methods section.

To identify genes that are differentially expressed in *S*. *coelicolor* WT and ∆*hsr* under oxidative stressing conditions, the recorded RPKM values of each gene within the respective transcriptome were compared. At first, the transcriptomes of the stressed and non-stressed WT were compared. The signal intensity ratio (M) / signal intensity (A) plots deduced from differential gene expression analysis are shown in Fig 3A. Similar comparisons were done with the transcriptomes of the stressed and non-stressed ∆*hsr* strain (Fig 3B). Genes with M-values ≥ +1.5 or ≤ −1.5 were considered to be differentially transcribed. While 28 up-regulated and 26 down-regulated genes were recorded in the WT strain (Fig 3A), no up-regulated and 20 down-regulated genes were recorded in the ∆*hsr* mutant (Fig 3B). These data indicate that all 28 up-regulated (M ≥ +1.5) genes in the WT (Table 1) are deregulated in the ∆*hsr* mutant.

**Table 1:** Up-regulated genes from the WT under oxidative stressing conditions.

SCO no.	Name	Function	M-value	Fold-change
SCO6094	*ssuC*	transport system integral membrane protein	3.19	9.13
SCO6095	*ssuB*	ABC transporter ATP-binding protein	3.81	14.03
SCO6096	*ssuA*	lipoprotein	4.05	16.56
SCO6097	*cysN*	sulfate adenylyltransferase subunit 1	3.91	15.03
SCO6098	*cysD*	sulfate adenylyltransferase subunit 2	3.4	10.56
SCO6099	*cysC*	adenylylsulfate kinase	3.51	11.39
SCO6100	*cysH*	phosphoadenosine phosphosulfate reductase	4.09	17.03
SCO6101		hypothetical protein	4.21	18.51
SCO6102	*cysI*	nitrite/sulfite reductase	3.22	9.32
SCO4164	*cysA*	thiosulfate sulfurtransferase	3.06	8.34
SCO4165		hypothetical protein	3.33	10.06
SCO2910	*cysM*	cysteine synthase	3.08	8.46
SCO2911		hypothetical protein	2.39	5.24
SCO2912		hypothetical protein	1.78	3.43
SCO4498		proton transport protein	3.62	12.30
SCO4499		TetR family transcriptional regulator	2.89	7.41
SCO6266		ScbA protein	3.24	9.45
SCO5772		hypothetical protein	3	8.00
SCO4881		polysaccharide biosynthesis-like protein	2.83	7.11
SCO3035		hypothetical protein	2.71	6.54
SCO1557^a^	*metQ*	lipoprotein, methionine transporter	1.18	2.26
SCO1558	*metI*	ABC transporter permease	1.81	3.51
SCO1559	*metN*	ABC transporter ATP-binding protein	2.39	5.24
SCO5958		ATP-binding protein, conbalt transport	1.66	3.19
SCO6124		hypothetical protein	1.75	3.36
SCO6045		hypothetical protein	1.59	3.01
SCO1968		hydrolase	1.59	3.01
SCO7549		unknown	1.51	2.85
SCO5485		small membrane protein	1.51	2.85
SCO1847^a^	*cobD*	cobalamin biosyntheis protein	1.39	2.63
SCO1848^a^	*cobQ*	cobyric acid synthase	1.42	2.67
SCO1849^a^	*cobN*	cobaltochelatase	1.07	2.10
SCO1850^a^	*chlD*	chelatase	0.96	1.943
SCO1851^a^	*cobO*	cob(I)alamin adenosyltransferase	0.64	1.6
SCO1852^a^	*cobI*	precorrin-2 C20-methyltransferase	0.87	1.83
SCO1553^a^	*cysG*	uroporphyrin-III C-methyltransferase	1.20	2.30
SCO1554^a^	*cobT*	nicotinate-nucleotide-dimethylbenzimidazole phosphoribosyltransferase	1.14	2.20
SCO5227^a^	*nrdX*	redoxin	1.45	2.73
SCO5226^a^	*nrdA*	ribonucleotide reductase large chain	1.45	2.73
SCO5225^a^	*nrdB*	ribonucleotide reductase small chain	1.30	2.46
SCO5224^a^		AraC-family transcriptional regulator	1.16	2.23
SCO7217^a^		ATP-binding protein, iron/heme/Cbl uptake	1.10	2.15

^a^Genes with M-values < 1.5

**Fig 4:**
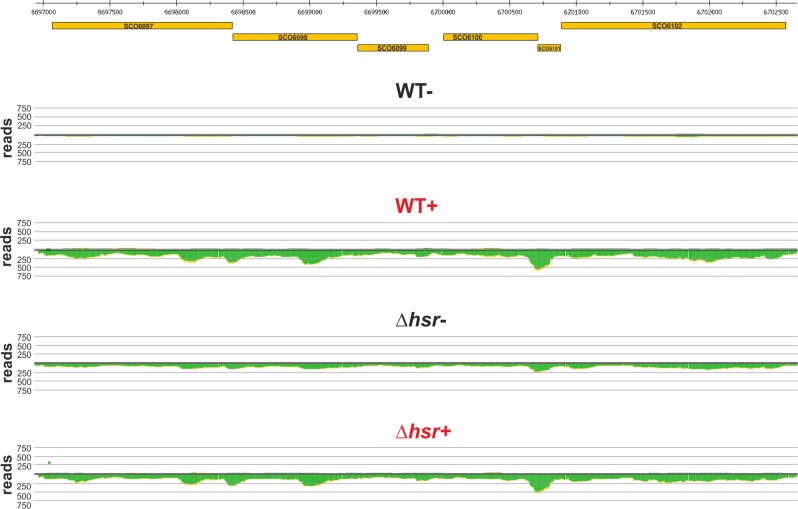
Transcriptional pattern of the sulfate-to-cysteine biosynthesis gene cluster. The figure shows the genomic position of the genes SCO6097-SCO6102 (orange boxes) and their transcriptional profile in *S*. *coelicolor* wild-type and *∆hsr* mutant under non- (WT- or *∆hsr*-) or oxidative-stressing (WT+ or *∆hsr*+, both written in red) conditions. The cumulated reads derived from primary transcripts are indicated with green color.

Among the up-regulated genes in the wild-type are those for sulfonate transport (SCO6094-SCO6096) and sulfate-to-cysteine biosynthesis (SCO6097-SCO6102, SCO4164 and SCO2910). Fig 4 shows the transcriptional pattern of the cluster SCO6097-SCO6102 in the WT as well as in the ∆*hsr* mutant. One can clearly see that transcriptional levels of the cluster are increased in the WT under oxidative-stressing conditions, whereas in the mutant a similar level of transcription is observed under non- and stressing conditions. The sulfur assimilation pathway for cysteine biosynthesis in *S*. *coelicolor* has been previously described in analogy to the one suggested in *Bacillus subtilis* [46, 47]. The pathway includes a stepwise reduction of sulfate to sulfide. The uptake of sulfate is predicted to be mediated by an unknown sulfate permease [47]. Sulfate is then converted to 3’-phosphoadenylyl sulfate (PAPS) by the concerted action of enzymes encoded by *cysN* (SCO6097), *cysD* (SCO6098) and *cysC* (SCO6099). The gene product of *cysH* (SCO6100) converts PAPS to sulfite. *cysA* (SCO4164) and *cysI* (SCO6102) encode sulfite reductase and thiosulfate sulfurtransferase, which catalyze the formation of sulfide and thiosulfate, respectively. Both metabolites are sulfur donors for incorporation of sulfur into O-acetyl-L-serine. This reaction is catalyzed by cysteine synthase encoded by *cysM* (SCO2910). The *cysA* gene (SCO4164) encoding thiosulfate sulfurtransferase is also related to the pathway for cysteine biosynthesis [48, 49]. In the absence of sulfate, *S*. *coelicolor* can utilize sulfonate as a sulfur source [50]. The uptake of sulfonate is likely mediated by the proteins encoded by the gene cluster *ssuABC* (SCO6094-SCO6096) [47].

Another up-regulated gene cluster (SCO1557-SCO1559) comprises the genes *metQ*, *metI* and *metN*, respectively. Although *metQ* shows an M-value of 1.2, we consider it as up-regulated. These genes are predicted to encode an uptake system for methionine, similar to one described in *E*. *coli* [51]. Interestingly, the gene SCO5958 encoding a putative ATP-binding protein involved in transport of cobalt is also up-regulated. This protein may be involved in the biosynthesis of cobalamin (see below).

**Fig 5:**
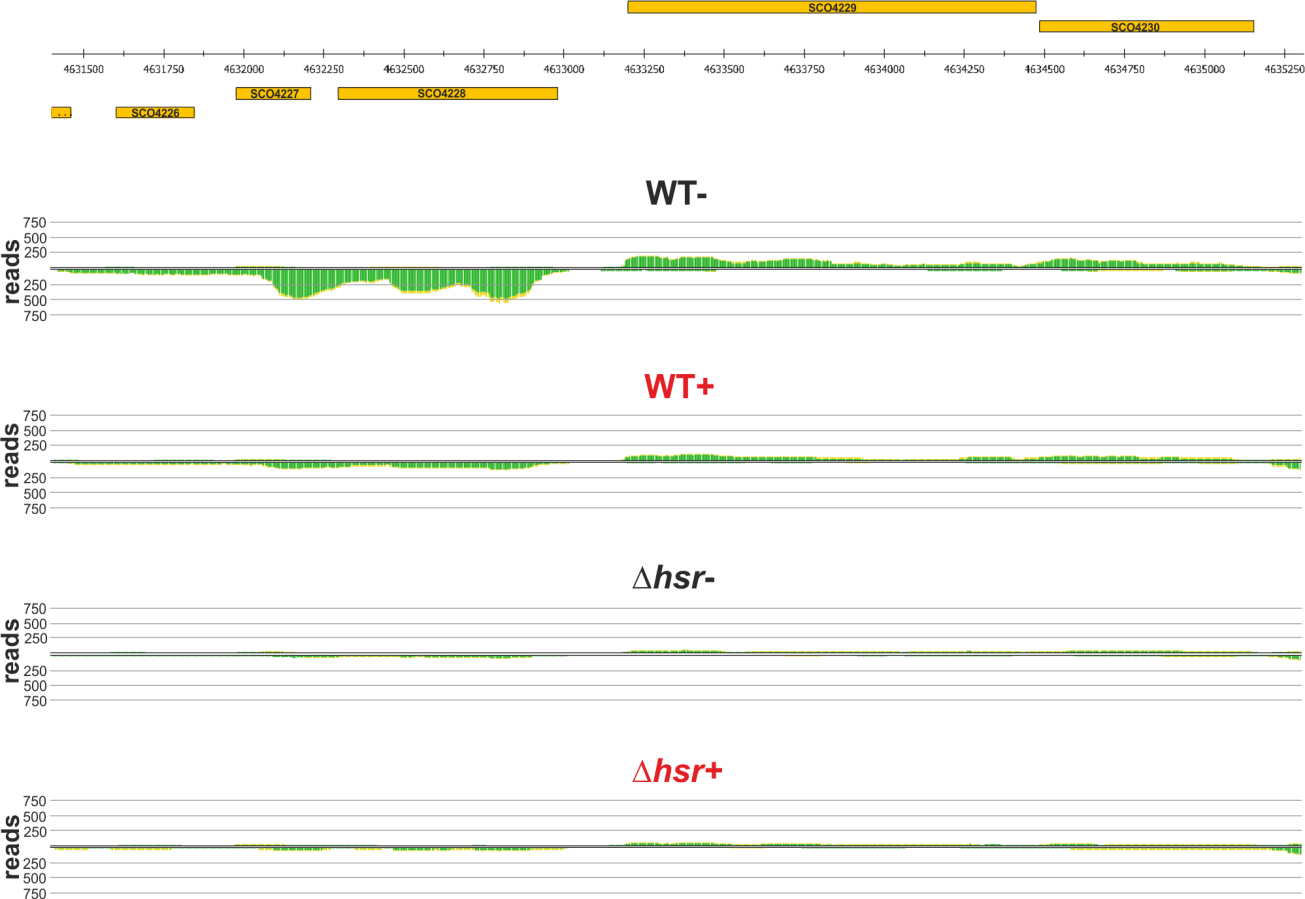
Transcriptional pattern of *phoRP* and neighboring genes. The figure shows the genomic position of the genes SCO4226-SCO4230 (orange boxes) and their transcriptional profile in *S*. *coelicolor* wild-type and *∆hsr* mutant under non- (WT- or *∆hsr*-) or oxidative-stressing (WT+ or *∆hsr*+, both written in red) conditions. The cumulated reads derived from primary transcripts are indicated with green color.

Looking for the down-regulated (M ≤ -1.5) genes, we noticed that from the 26 genes down-regulated in the wild-type 14 are deregulated in the ∆*hsr* mutant (Table 2). These include the two-component system (TCS) genes *phoR* (SCO4229) and *phoP* (SCO4230) as well as its accessory gene *phoU* (SCO4228). The PhoU-PhoR-PhoP system governs the transcription of phosphate-regulated genes in streptomycetes [52, 53]. The adjacent genes SCO4226 and SCO4227 are also down-regulated and were suggested to act in concert with PhoU-PhoR-PhoP [53]. Fig 5 shows the transcriptional pattern of the gene cluster SCO4226-SCO4230 in the WT as well as in the ∆*hsr* mutant. While in the wild-type the transcription is considerably down-regulated under oxidative-stressing conditions, in the mutant no clear differences were observed. Recently, the crystal structure of the protein encoded by SCO4226 was crystallized in complex with nickel [54]. PitH2, encoded by SCO1845, is a predicted low-affinity phosphate transport protein. This gene as well as SCO4226 and SCO4227 are regulated by the TCS PhoRP [53, 55]. Further down-regulated genes are *hpxQ* (SCO6209), *hpxT* (SCO6210) and *hpxO* (SCO6211). Although *hpxT* shows an M-value of -1.3, we consider it as down-regulated (Table 2). HpxQ is 2-oxo-4-hydroxy-4-carboxy-5-ureidoimidazoline (OHCU) decarboxylase, HpxT a 5-hydroxylisourate (HIU) hydrolase and HpxO an urate oxidase. These enzymes catalyze the sequential conversion of urate to allantoin [56]. Urate is an efficient scavenger of singlet oxygen and radicals [57]. Thus, its presence will have a positive effect on conditions of oxidative stress.

**Table 2:** Down-regulated genes in the WT, but deregulated in the *∆hsr* mutant.

SCO no.	Name	Function	M-value	Fold-change
SCO4226		nickel-binding protein	-2.03	0.24
SCO4227		hypothetical protein	-2.86	0.14
SCO4228	*phoU*	phosphate transport system regulator	-3.04	0.12
SCO4229	*phoR*	sensor kinase	-2.13	0.23
SCO4230	*phoP*	response regulator	-1.83	0.28
SCO6209	*hpxQ*	OHCU decarboxylase	-1.57	0.34
SCO6210^a^	*hpxT*	hydroxyisourate hydrolase	-1.30	0.41
SCO6211	*hpxO*	urate oxidase	-1.81	0.29
SCO1845	*pitH2*	low-affinity phosphate transport protein	-2.01	0.25
SCO6771		small hydrophobic hypothetical protein	-1.84	0.28
SCO6702		3-oxoadipate CoA-transferase subunit B	-1.7	0.31
SCO1541		putative regulator	-1.7	0.31
SCO7608		hypothetical protein	-1.64	0.32
SCO4440		hypothetical protein	-1.58	0.33
SCO4353		hypothetical protein	-1.57	0.34

^a^Gene with an M-value < -1.5

### Other differentially expressed genes

We previously reported that the HbpS protein from *S*. *reticuli* binds the tetrapyrrolic compounds heme and aquo-cobalamin (vitamin B_12a_) [17, 44]. Given that HbpS modulates the function of HbpS-SenS-SenR, we additionally checked if genes related to heme or cobalamin are differentially expressed. We focused the analysis on genes that are up/down-regulated in the wild-type, but deregulated in the ∆*hsr* mutant. We did not detect differentially expressed genes with M ≥ 1.5 or M ≤ -1.5, but with M values close to 1.5 (Table 1). This is the case for genes involved in synthesis of the corrin ring of cobalamin (SCO1847-SCO1853) (S1 Fig), uroporphyrin-III methyltransferase (SCO1553, named *cysG*) and nicotinate-nucleotide-dimethylbenzimidazole phosphoribosyltranferase (SCO1554, named *cobT*). CysG is predicted to catalyse the S-adenosylmethionine-dependent methylation of uroporphyrinogen III, leading to the product sirohydrochlorin that is the common precursor of both siroheme and cobalamin (vitamin B_12_) [58]. CobT has been shown to be involved in the synthesis and assembly of the nucleotide loop of cobalamin [59]. The increased synthesis of cobalamin will be beneficial for cells under oxidative stressing conditions, as this tetrapyrrole is an efficient antioxidant [60]. Moreover, the transcription of the genes *nrdA* (SCO5226), *nrdB* (SCO5225) and *nrdS* (SCO5224) is up-regulated (Table 1). NrdAB is a ribonucleotide reductase (RNR) class Ia that supports DNA synthesis and repair. Interestingly, the synthesis of NrdAB and NrdS (AraC-like regulator) is regulated by a cobalamin-binding riboswitch [61]. The genes SCO5226-SCO5224 are co-transcribed with SCO5227 that encodes a redoxin-like protein. Redoxin domains are found in peroxiredoxin, thioredoxin and glutaredoxin proteins, and thus, they are thought to play a large role in anti-oxidant defense [62]. The last identified gene belonging to this group is SCO7217 (M = 1.1) that encodes a predicted ATP-binding component of a transport system. SCO7217 is co-transcribed with SCO7216 and SCO7218 that encode a putative FecCD-family membrane transport protein and a substrate-binding lipoprotein, respectively. Homologues to these three proteins together form an ABC transport system for the uptake of iron-complexes or other related compounds such as heme and cobalamin [63].

Given that mycothiol is the major thiol found in streptomycetes as well as in other actinomycetes [25], we checked whether *mshA* (the first gene in the biosynthetic pathway of mycothiol in *S*. *coelicolor*) is differentially expressed. *mshA* (SCO4204) shows an M-value of -0.04 in the wild-type and 0.1 in the ∆*hsr* mutant. This indicates that *mshA* is not differentially expressed neither in the wild-type nor in the mutant, und thus, its transcription is not HbpSc-SenSc-SenRc-dependent. This is in agreement with previous works which showed that the transcription of *mshA* is under the control of the sigma factor σ^R^ [64].

The biochemical pathways, in which the mentioned up- and down-regulated genes may be involved, are closely related to the response of the cell to oxidative stress. This is in line with the redox-sensing role of HbpSc-SenSc-SenRc.

### Validation of RNA-Seq results by qRT-PCR

**Fig 6:**
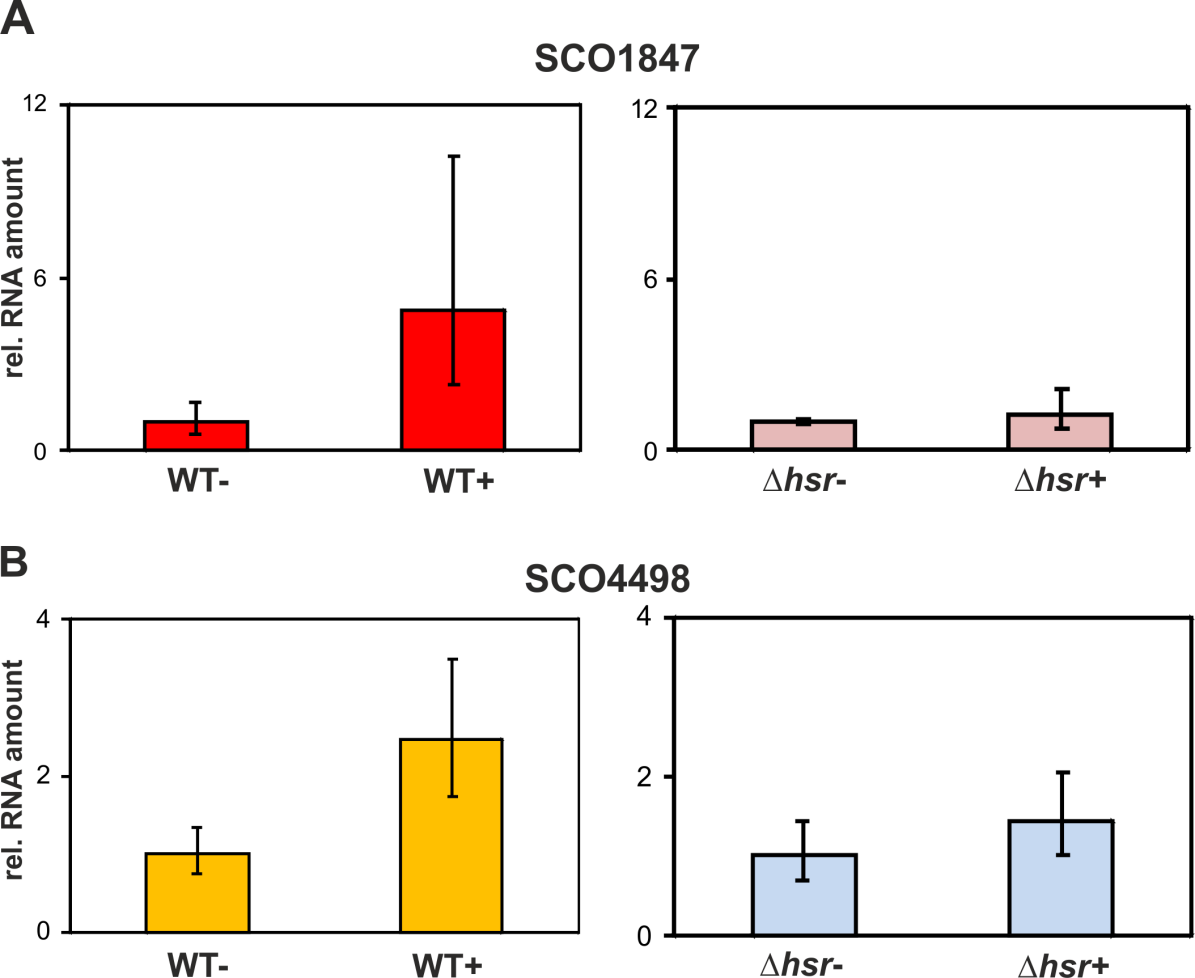
qRT-PCR analysis of SCO1847 and SCO4498. Total RNA of *S*. *coelicolor* wild-type and *∆hsr* mutant cultivated under under non- (WT- or ∆hsr-) or oxidative-stressing (WT+ or ∆hsr+) were used to quantify the amount of SCO1847 (A) and SCO4498 (B) transcripts by qRT-PCR. Primers used are indicated in S1 Table. The Y-axis indicates the relative amount of primary transcripts (rel. RNA amount).

To validate the RNA-Seq results by qRT-PCR, we randomly selected two (SCO1847 and SCO4498) from the group of genes described in Tables 1 and 2. Total RNA was obtained from three biological replicates that were cultivated under identical physiological conditions as used to obtain RNA for RNA-Seq. Preparation of samples, reactions and calculations were done as described in the Materials and Methods section. While in the wild-type the relative amount of SCO1847 (Fig 6A) and SCO4498 (Fig 6B) transcripts is enhanced (5- and 2.5-fold, respectively) on conditions of oxidative stress, in the mutant no significant changes were observed. This is in line with the RNA-Seq data showing that the transcription on both genes is deregulated in the ∆*hsr* mutant. The fold-changes recorded by RNA-Seq and qRT-PCR in the wild-type, however, differ. Whereas for SCO1847 the fold-change by RNA-Seq (3-fold) and qRT-PCR (5-fold) moderately differs, the fold-change for SCO4498 recorded by RNA-Seq (11-fold) considerably differs to that obtained by qRT-PCR (2.5-fold). There are several reasons which might explain this difference. RNA-Seq provides often a greater nucleotide level resolution that allows an accurate quantification of expression levels of entire genes [65]. Additionally, primer design and other experimental conditions (i.e. amplification of GC-rich DNA) are probably responsible for varying expression results by qRT-PCR compared to RNA-Seq.

### SenRc specifically interacts with regulatory DNA regions of candidate genes

**Fig 7:**
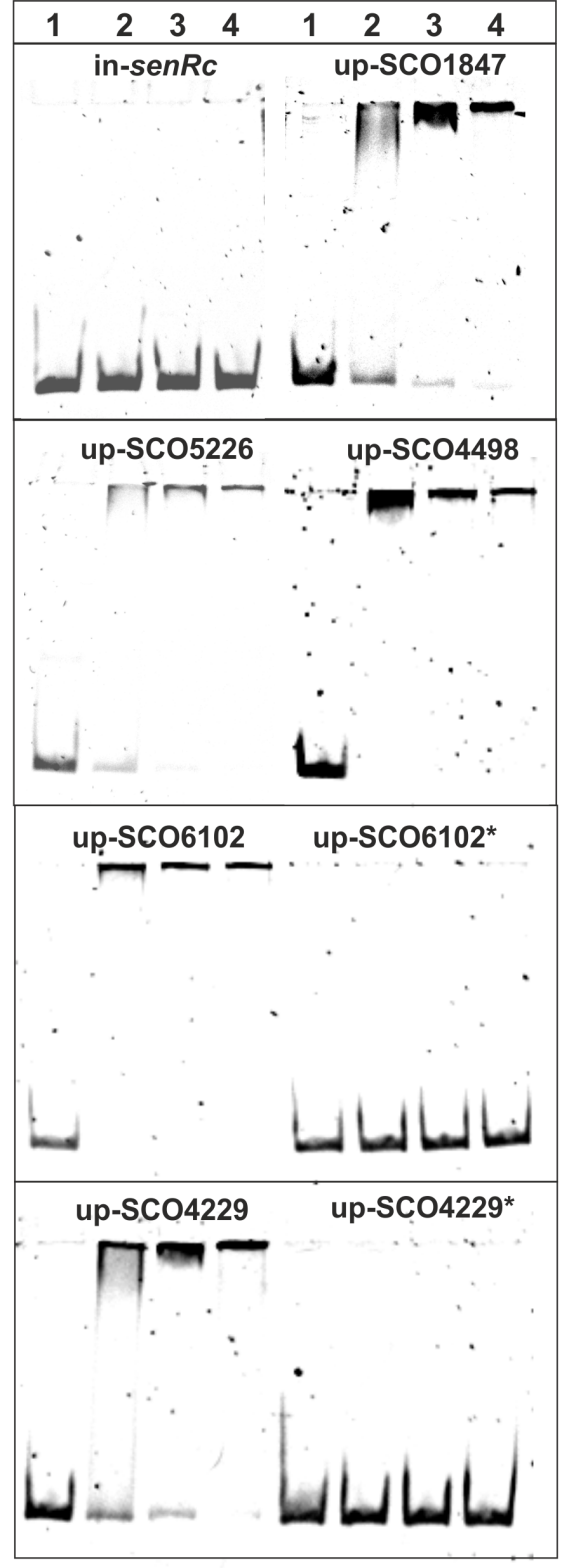
EMSAs with the isolated regulator SenRc. 20 ng of DNA fragments (~200 bp) consisting of the upstream region of the indicated genes were incubated without (lane 1) or with increasing amounts (0.5 μg, lane 2; 1 μg, lane 3 and 2 μg, lane 4) of isolated SenRc in DNA-binding buffer at 30°C for 15 min. The DNA fragment comprising the internal region (~200 bp) of *senRc* (in-*senRc*) was used as a control. To amplify DNA fragments, primers listed in S1 Table were used. Analyses were performed with 7% polyacrylamide gels.

RNA-Seq and qRT-PCR results showed the transcription of several genes might be directly or indirectly controlled by the HbpSc-SenSc-SenRc system. To get more insights, we cloned, expressed, and purified SenRc as His-tag fusion protein (S2 Fig) that was used for electrophoretical mobility shift assays (EMSAs). The upstrean DNA regions (~200 bp) of four up-regulated (SCO1847, SCO5226, SCO4498 and SCO6102) and one (SCO4229) down-regulated genes were obtained by PCR and purified using PCR cleanup kits. For PCR, chromosomal DNA of *S*. *coelicolor* and primers listed in S1 Table were used. For a control experiment, a 200 bp DNA fragment comprising the coding region of the *senRc* gene was used. EMSAs using the control DNA showed no binding of SenRc. However, under identical conditions SenRc was able to interact with the DNA upstream of the studied genes (Fig 7). EMSAs were repeated three times. The outcome was always the same.

Previous DNaseI footprinting studies using the SenR protein from *S*. *reticuli* allowed to identify three different SenR DNA-binding sites (I-III) [66]. The *S*. *reticuli* SenR and SenRc from *S*. *coelicolor* A3(2) contain a C-terminally located helix-turn-helix DNA-binding motif. The motifs have 86% sequence identy (not shown). Thus, we expected that *S*. *coelicolor* SenRc may have similar DNA-binding site(s) as those described for the *S*. *reticuli* SenR protein. To simplify the analysis, we checked the sequences within the regulatory regions of those genes or gene clusters, whose functions are described in the previous paragraphs. Alignments were done using Clustal Omega [67]. Comparisons using the three SenR DNA-binding sites and the promoter regions of the studied genes allowed the identification of DNA sequences that display different degrees (from 53 to 91%) of identity to just the binding site III (Table 3). Some degree of variation in the sequence conservation within operators of a transcriptional regulator is not surprising and it was reported, for example, for the PhoB protein from *S*. *coelicolor* [68]. In addition to identical bases, the recorded putative binding sites upstream of the genes SCO4498 and SCO2912 contain direct repeats (Table 3). The exact role of the repeats in SenRc-DNA interactions remains, however, to be elucidated. Other transcriptional regulators, such as XylR from *E*. *coli*, interact with directed repeats that are located within their DNA-binding regions [69].

**Table 3:** Predicted DNA-binding sites of SenRc.

	Sequence (5‘- to -3‘)^a^	Distance to start codon	% identity^b^
*S*. *reticuli* SenR binding site III	CCGGGCCGCGTCCCGT	106	100
Predicted SenRc DNA-binding sites in the upstream DNA regions of the indicated genes			
SCO1845	GGCCGCG**A**CCC	96	91
SCO6102	CCGGGCCGCT	152	90
SCO6209	CC**A**GCCGC**C**GTCC**G**GT	51	87
SCO1559	CCG**T**GCCGCGT**T**CCG	31	87
SCO4498^c^	***CGCGTC***CC**ATC**CCGCGTCCCGT	13	81
SCO5958	CCGGGC**G**GCG**CGGC**TCC**A**GT	142	81
SCO4229	GGGCCG**GA**C**G**G**T**TC**G**CCGT	15	79
SCO4164	CCG**C**G**G**C**A**CG**G**CCCG	55	73
SCO2912^c^	***GTTCC*A*GTTCC*G**C**T**CGT***T***CCG	86	73
SCO1554	CGG**TG**CG**C**CGTCCCG	47	73
SCO1847	CCGGCGCG**G**TCCCG	167	71
SCO5226	CC**CTCC**GCCG**G**G**C**CCCGT	22	69
SCO5227	C**AC**GGCCGCG**G**C**A**C**AC**	62	63
SCO7217	C**GACAT**C**CT**GTCCCG	107	53

^a^ The *S*. *reticuli* SenR binding site III as well as conserved bases in the predicted SenRc-binding sites are marked with a grey background

^b^ % of identity between the indicated sequence and the *S*. *reticuli* SenR binding site III

^c^ Directed repeats within or close to the predicted SenRc-binding sites in front of SCO4498 and SCO2912 are written in italics

**Fig 8:**
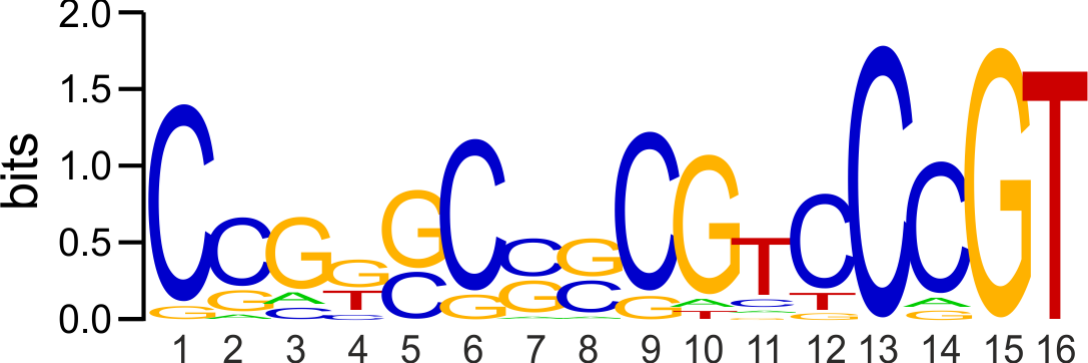
Conservation of nucleotides within the predicted SenRc-binding sites. The *S*. *reticuli* SenR DNA-binding site III as well as the sites predicted to interact with SenRc (Table 3) was used for the alignment. The depicted sequence logo was created with WebLogo.

To get a better view, we aligned the predicted SenRc DNA-binding sites and the *S*. *reticuli* SenR DNA-binding site III with each other using WebLogo [70]. The deduced consensus sequence contains an imperfect inverted repeat (CGGGCCGCGTCCCG) (Fig 8). The alignment additionally shows that positions 13, 15 and 16 (bits >1.5) as well as 1, 6, 9, 10 and 14 (bits between 1–1.5) are highly conserved. Binding-site positions with conservation higher than 1 bit are expected to be located at the major groove of the DNA [71]. Thus, the conserved bases within the predicted DNA-binding sites may directly interact with SenRc.

To check whether the predicted binding sites are involved in the interaction with SenRc, we deleted them in front of SCO6102 (up-SCO6102* in Fig 7) and SCO4229 (up-SCO4229* in Fig 7) by insertion of a randomly selected sequence of the same length. EMSAs using the mutated DNA showed no interaction with SenRc.

These data suggest that the response regulator SenRc may directly regulate the transcription of the studied genes by binding to their promoter regions.

## Discussion

Our previous biochemical and biophysical studies allowed a detailed characterization of the interaction between the secreted protein HbpS, the membrane sensor kinase SenS and the cytoplasmic response regulator SenR from *S*. *reticuli*. We additionally characterized redox stress-based post-translational modifications of these proteins that form a novel type of redox-signaling system and is widespread in different actinobacteria. In this study, we used transcriptomics to identify genes that are under the control of HbpS-SenS-SenR. Since *S*. *coelicolor* is the model streptomycete, we focused the investigations on the homologous system, named here HbpSc-SenSc-SenRc. Mutational studies coupled with growth assays showed that HbpSc-SenSc-SenRc provides *S*. *coelicolor* with an increased resistance to the hazardous effects of oxidative stress. RNA-Seq profiling showed that under oxidative stress, the transcription of genes involved in synthesis of cysteine and cobalamin (vitamin B_12_) as well as in the uptake of methionine are up-regulated in the wild-type, but deregulated in the ∆*hsr* mutant. Cysteine and methionine are sulfur-containing amino acids that are good consumers of ROS, which are electron-deficient and tend to rapidly react with both amino acids [72]. Thus, both can function as antioxidants within proteins by protecting other amino acids that are crucial for protein function [73, 74]. Additionally, cysteine is well-known for its capability to function as redox buffer, thereby together with other low-molecular-weight thiols (i.e. mycothiol) or small redox proteins (i.e. thioredoxin) ensuring a reducing environment in the cell [25, 75]. Interestingly, the transcription of the SCO5227 gene that encodes a redoxin-like protein is also up-regulated in the wild-type under oxidative stressing conditions. This redoxin-like protein contains seven cysteine residues, two of them are located within a CXXC motif that as in other redoxins might be essential for reducing disulfide bonds of target proteins and maintaining intracellular redox homeostasis [62, 76]. Another compound that can act as a redox buffer is urate. Urate is generated from the metabolism of purines and is an efficient scavenger of singlet oxygen and radicals [57]. Notably, the transcription of the *hpxQ*, *hpxT* and *hpxO* genes, whose products participate in the degradation of urate [56], is down-regulated in the wild-type in a HbpSc-SenSc-SenRc-dependent fashion. Thus, blocking of urate degradation will have a positive effect on conditions of oxidative stress.

HbpSc-SenSc-SenRc seems to interplay with the regulator NdgR that is necessary for the thiol oxidative stress response in *S*. *coelicolor* [49]. ChIP-Seq experiments revealed that NdgR binds the regulatory DNA regions of the genes for sulfate-to-cysteine biosynthesis (SCO6097-SCO6102, SCO4164 and SCO2910). Subsequent studies showed that the transcription of these genes is elevated under oxidative stressing conditions, but this was not NdgR-dependent [49]. The authors suggested that there must be another regulatory system controlling this transcription. This is in line with our RNA-Seq data which clearly show that the transcription of the sulfate-to-cysteine biosynthesis genes is HbpSc-SenSc-SenRc-dependent. The transcriptomics data additionally suggested that the HbpSc-SenSc-SenRc signaling pathway is likely linked to the PhoRP pathway. This two-component system governs the transcription of phosphate-regulated genes in streptomycetes [52]. The transcription of *phoRP* as well as of the adjacent genes *phoU*, SCO4226 and SCO4227 is down-regulated in a HbpSc-SenSc-SenRc-dependent manner. This is also the case for the transcription of SCO1845 encoding a putative low-affinity phosphate transport that has been shown to be also controlled by PhoRP [77]. Phosphate is highly relevant in cells, as it is a component of nucleotides that serve as energy storage (i.e. ATP and GTP) or as backbone of nucleic acids. Phosphate is additionally an important component of membrane phospholipids and is required for post-translational modifications in proteins [78, 79]. In addition to phosphate assimilation, PhoRP has been shown to be involved in the response to oxidative stress [53, 77]. The studies showed that the synthesis of the major *S*. *coelicolor* vegetative catalase CatA and of the catalase-peroxidase CpeB is positively regulated by PhoRP, but likely not directly [77]. The HbpSc-SenSc-SenRc-mediated down-regulation of PhoRP would imply a low synthesis of CatA and CpeB under the experimental conditions used in this work.

What is the relationship between oxidative stress and enhanced production of cobalamin? Suarez-Moreira, Yun [80] showed that cobalamin (vitamin B_12_) can scavenge the highly reactive superoxide anion. Thus, it seems that cobalamins play an important role in the overall anti-oxidative stress response in cells. In humans, the deficiency of cobalamin has been implicated in different disorders [81]. Usually, cobalamins act as co-factors for mutases, dehydratases, deaminases, ribonucleotide reductases, methyl transferases, methionine synthases and methylmalonyl-CoA-mutases [82]. It is worth noting that streptomycetes belong to the relatively small group of bacteria that are able to *de novo* synthesize cobalamin [83]. In streptomycetes there are Cbl-dependent enzymes that catalyse a set of modifications to chemical backbones during the biosynthesis of antibiotics [84, 85]. Recently, cobalamin was shown to act as a co-factor of transcriptional regulators in *Rhodobacter* and *Myxococcus*. It was suggested that an altered oxidation state of the cobalt ion in cobalamin or its derivatives leads to structural changes in the regulatory proteins [86–88]. We recently showed that the *Streptomyces* HbpS protein binds aquo-cobalamin with low affinity suggesting that this cobalamin might be a co-factor during the HbpS-mediated signaling pathway [17]. We aditionally showed that oxidative modifications in HbpS led to conformational changes in the protein, resulting in activation of SenS autophosphorylation [22, 23]. The unmodified form of HbpS, however, inhibits SenS autophosphorylation. We suggest that the elevated synthesis of cobalamin has an anti-oxidative effect within the *Streptomyces* mycelia and at the same time the bound cobalamin at HbpS might protect the protein from oxidation by scavenging ROS. Future efforts are, however, necessary to exactly characterize the interplay of these processes.

Looking further afield, it has been reported that adenosylcobalamin (AdoCbl; coenzyme B_12_) interacts with the so-called B_12_ riboswitch in front of the *S*. *coelicolor nrd*ABS (SCO5526-SCO5524) operon to regulate its transcription [61]. Our transcriptomics data showed that *nrdABS* is up-regulated in a HbpSc-SenSc-SenRc-manner under oxidative-stress conditions. NdrAB is a class Ia ribonucleotide reductase (RNR) that, together with the class II RNR NdrJ, supports DNA synthesis and repair in *S*. *coelicolor* [89]. Since the side effects of oxidative stress induce DNA mutations [90], an increased production of NdrAB is certainly highly relevant in anti-oxidative stress response processes. NdrJ is AdoCbl-dependent and the primary RNR system during vegetative growth. It has been suggested that NdrAB might function under certain conditions when NdrJ is inactive, for example, when AdoCbl is not available [61]. Sequence comparisons using the *S*. *coelicolor* genome allowed the identification of putative B_12_ riboswitches in the 5’-untranslated region (UTR) of eight additional genes or gene clusters [61]. Interestingly, the transcription of three of them (SCO1847-1853, SCO5958 and SCO7217) is up-regulated in response to oxidative stress in a HbpSc-SenSc-SenRc-dependent manner (Table 1). The gene products are related to cobalamin synthesis (SCO1847-1853), transport of cobalt and/or cobalamin (SCO5958), and transport of iron and/or heme and/or cobalamin (SCO7217). We suggest that the increased synthesis and uptake of cobalamin will provide the cell with sufficient levels of this compound not only for anti-oxidative defense, but also as co-factor for Cbl-dependent enzymes such as NdrJ.

We showed that the regulator SenRc binds among others the upstream region of the *nrdA* gene (up-SCO5226 in Fig 7). Sequence analysis indicates that the putative binding site is located near to the DNA region encoding the B_12_ riboswitch (not shown). The RNA-Seq data additionally suggest that the response regulator SenRc negatively regulates the transcription of the operon comprising *ndrA* under non-stressing conditions. This would imply that SenRc and the B_12_ riboswitch might act in concert during transcriptional repression. Interestingly, nine of the identified B_12_ riboswitches in *S*. *coelicolor* [61] are located in the 5’-UTR region of four genes/gene clusters that are regulated by SenRc. A B_12_ riboswitch in *Listeria monocytogenes* and *Enterococcus faecalis* has been shown to act in concert with the two-component response regulator EutV that regulates the transcription of *eut* genes, whose products enable ethalomine utilization and require coenzyme B_12_ as co-factor. In the absence of coenzyme B_12_, EutV binds the RNA elements, that build the riboswitch, and thereby prevents transcriptional termination of the *eut* genes. In the presence of coenzyme B_12_, EutV cannot bind the RNA elements, leading to the expression of *eut* genes [91, 92]. Future efforts should clarify whether SenRc and the *Streptomyces* B_12_ riboswitch physically interact and the physiological relevance of the interaction.

The transcriptomics data indicate that the HbpSc-SenSc-SenRc system from *S*. *coelicolor* positively influences the synthesis of compounds acting as redox buffers or scavengers of ROS. We did not record an elevated synthesis of scavenging enzymes such as catalases or superoxide dismutases. Previously, we have, however, shown that the HbpS-SenS-SenR system from *S*. *reticuli* controls the transcription of the catalase-peroxidase *cpeB* gene [45]. We interpret these differences as following. CpeB as well as HbpS-SenS-SenR was discovered during the cultivation of *S*. *reticuli* in presence of crystalline cellulose [93]. During breakdown of cellulose ROS are produced, and thus, an elevated synthesis of CpeB might help to protect the *S*. *reticuli* mycelia from ROS. *S*. *coelicolor* is not able to degrade crystalline cellulose (not shown), suggesting the link between CpeBc and HbpSc-SenSc-SenRc to be unnecessary. Moreover, *cpeB* and *hbpS-senS-senR* are clustered on the *S*. *reticuli* genome, but not in *S*. *coelicolor*. As outlined in the introduction section, homologous genes to *hbpS-senS-senR* are found in the same relative transcriptional orientation on different actinobacterial genomes. Remarkably, most of these homologues including that of *S*. *coelicolor* are not clustered with a *cpeB*-like gene (Fig 1). This may indicate that CpeB and HbpS-SenS-SenR act in concert most likely only in *S*. *reticuli* and other few *Streptomyces sp*. The main role of all HbpS-SenS-SenR-like systems, however, may be the same: sensing and response to oxidative stress.

## Conclusions

The redox-sensing system HbpSc-SenSc-SenRc from *S*. *coelicolor* provides this bacterium with an efficient defense mechanism under conditions of oxidative stress. The transcriptomics data suggest that this system controls the non-enzymatic response of *S*. *coelicolor* to counteract the hazardous effects of oxidative stress. Binding of the response regulator SenRc to regulatory regions of some of the studied genes indicates that the regulation is direct. We emphasize, however, that HbpSc-SenSc-SenRc may act in concert with other regulatory modules such as transcriptional regulators, two-component systems and riboswitches. We additionally expect that the redox-active metabolites heme and cobalamin play a significant role in these signaling events. The interplay might guarantee the fine-tuning of sensing and response to oxidative stress. Regulatory networks in streptomycetes as well as in other organisms are highly dynamic and can encompass overlapping signaling cascades to link diverse aspects of growth, morphology, and secondary metabolite production, pathways in which oxidative stress might occur.

## Supporting Information

S1 Fig Transcriptional pattern of genes involved in cobalamin synthesis. The figure shows the genomic position of the genes SCO1847-SCO1853 (orange boxes) and their transcriptional profile in *S*. *coelicolor* wild-type and *∆hsr* mutant under non- (WT- or *∆hsr*-) or oxidative-stressing (WT+ or *∆hsr*+) conditions. The cumulated reads derived from primary transcripts are indicated with green color.
(DOCX)

S2 Fig Isolated SenRc. SenRc was isolated as His-tag fusion protein by Ni^2+^-NTA affinity chromatography. An aliquot of the eluate containing 10 μg protein (lane 1) was analyzed by SDS-PAGE. Protein markers (lane M) were also loaded into the polyacrylamide gel. After electrophoresis the proteins were stained using PageBlue. Their molecular weigth is indicated. The arrow indicates the observed SenRc protein band.
(DOCX)

S1 Table List of primers used in this work. (DOCX)

S2 Table Summary of sequencing and mapping statistics. (DOCX)
