# Supplementary material for: Deciphering the Transcriptional Response Mediated by the Redox-Sensing System HbpS-SenS-SenR from Streptomycetes
Source: PLoS One. 2016 Aug 19;11(8):e0159873. doi: 10.1371/journal.pone.0159873 (PMC4991794; doi:10.1371/journal.pone.0159873)
Supplement: S1 Fig — The figure shows the genomic position of the genes SCO1847-SCO1853 (orange boxes) and their transcriptional profile in S. coelicolor wild-type and ∆hsr mutant under non- (WT- or ∆hsr-) or oxidative-stressing (WT+ or ∆hsr+) conditions. The cumulated reads derived from primary transcripts are indicated with green color. (DOCX) [file pone.0159873.s001.docx]

**
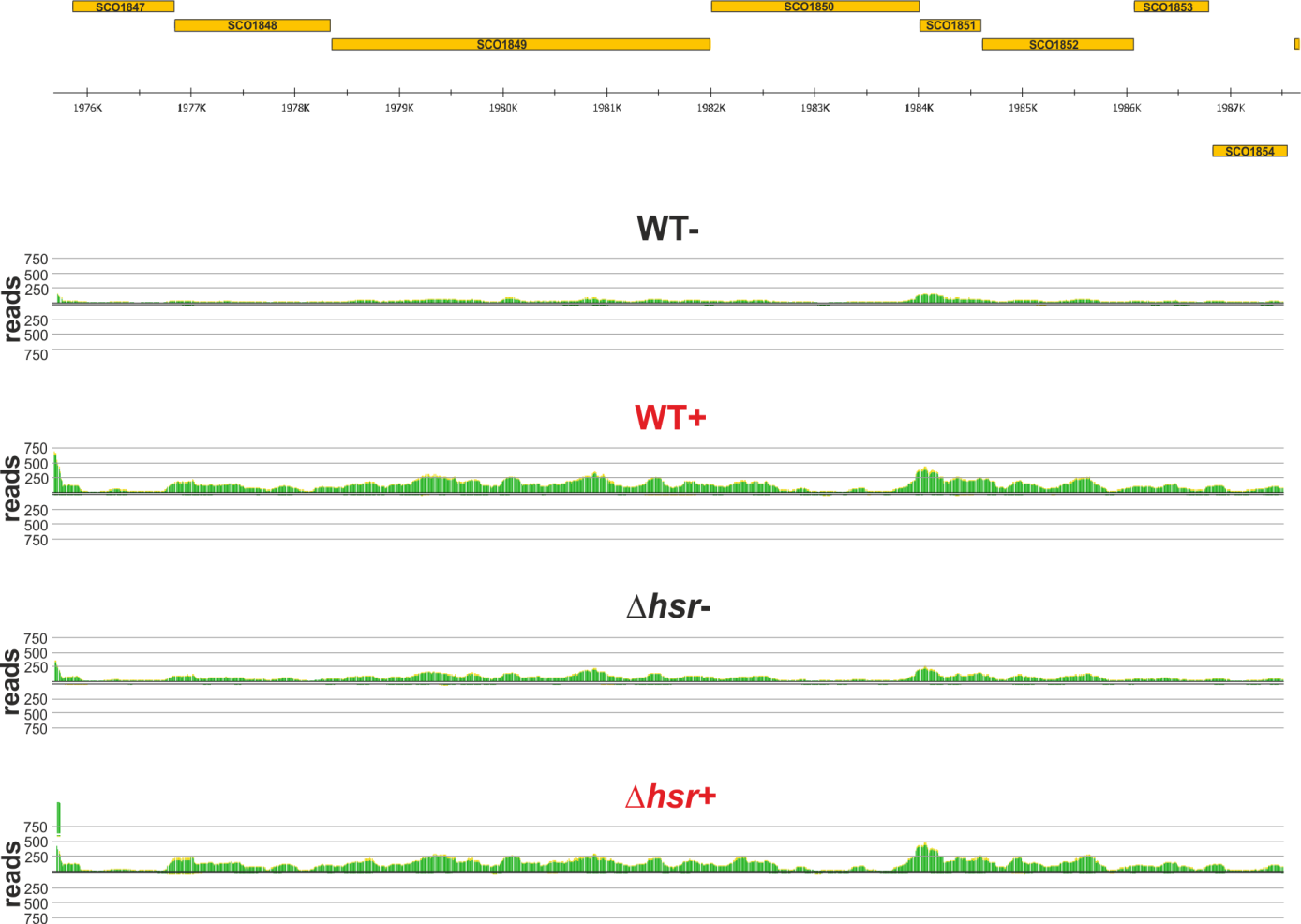
**

**S1 Figure. Transcriptional pattern of genes involved in cobalamin synthesis.** The figure shows the genomic position of the genes SCO1847-SCO1853 (orange boxes) and their transcriptional profile in *S. coelicolor* wild-type and *∆hsr* mutant under non- (WT- or *∆hsr*-) or oxidative-stressing (WT+ or *∆hsr*+) conditions. The cumulated reads derived from primary transcripts are indicated with green color.
