# Supplementary material for: Deciphering the Transcriptional Response Mediated by the Redox-Sensing System HbpS-SenS-SenR from Streptomycetes
Source: PLoS One. 2016 Aug 19;11(8):e0159873. doi: 10.1371/journal.pone.0159873 (PMC4991794; doi:10.1371/journal.pone.0159873)
Supplement: S2 Fig — SenRc was isolated as His-tag fusion protein by Ni2+-NTA affinity chromatography. An aliquot of the eluate containing 10 μg protein (lane 1) was analyzed by SDS-PAGE. Protein markers (lane M) were also loaded into the polyacrylamide gel. After electrophoresis the proteins were stained using PageBlue. Their molecular weigth is indicated. The arrow indicates the observed SenRc protein band. (DOCX) [file pone.0159873.s002.docx]

**
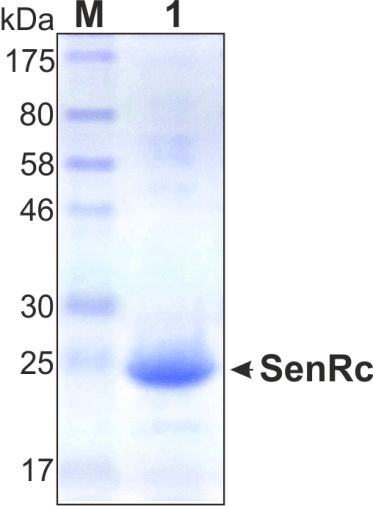
**

**S2 Figure. Isolated SenRc.** SenRc was isolated as His-tag fusion protein by Ni^2+^-NTA affinity chromatography. An aliquot of the eluate containing 10 µg protein (lane 1) was analyzed by SDS-PAGE. Protein markers (lane M) were also loaded onto the polyacrylamide gel. After electrophoresis the proteins were stained using PageBlue. Their molecular weigth is indicated. The arrow indicates the observed SenRc protein band.
