# Supplementary material for: Deciphering the Transcriptional Response Mediated by the Redox-Sensing System HbpS-SenS-SenR from Streptomycetes
Source: PLoS One. 2016 Aug 19;11(8):e0159873. doi: 10.1371/journal.pone.0159873 (PMC4991794; doi:10.1371/journal.pone.0159873)
Supplement: S1 Table — (DOCX) [file pone.0159873.s003.docx]

**S1 Table. List of primers used in this work**

| **Designation** | **Sequence (5‘ → 3‘)** | | **Property/use** |
| --- | --- | --- | --- |
| **Primers for cloning** | | | |
| LHinfor | CGGAAGCTTGGCCGGTTCTGGGTCTCC | | *Hin*dIII site underlined |
| LPstrev | CTACTGCAGGCCCGGCCCGCCGCCTC | | *Pst*I site underlined |
| RBamfor | GGAGGATCCGACCCGACCCGACCCTG | | *Bam*HI site underlined |
| RKpnrev | CACGGTACCCAGGGCCTCGGTCCGCGC | | *Kpn*I site underlined |
| PKpnFor | CCGGGCGGTACCTAGCGCAGCGCC | | *Kpn*I site underlined |
| PBamRev | GCGCGAGGATCCACGGGGTGCGGGG | | *Bam*HI site underlined |
| PBamFor | CCCCGCACCCCGTGGATCCTCGCGC | | *Bam*HI site underlined |
| PHinRev | GCGAAGCTTCGGGTCGGGGCGTCACG | | *Hin*dIII site underlined |
| PRForNco | GCCGCCATGGCCACCGACCCCGCCGCCCG | | *Nco*I site underlined |
| PRRevHind | GTCAAGCTTTCACGGCAGCAGGCGCTG | | *Hin*dIII site underlined |
| **Primers for qRT-PCR** | | | |
| RT1847for | CCGCCGCCGGCCTCCTCGGTGACC | | To amplify SCO1847 |
| RT1847rev | GCACCCGTTCCACGGCGCCCGCGG | | To amplify SCO1847 |
| RT4498for | CTGGGCCCAGATACTCGCCGGTCTC | | To amplify SCO4498 |
| RT4498fev | GACCTTCTCCAGGGTGGTGACCAG | | To amplify SCO4498 |
| **Primers for EMSA** | | | |
| SRinfor | CACCGGATACCTGTTGAAGGCCGAAC | To amplify in-*senRc* | |
| SRinrev | CTGATGAACAGGGCGCGGGCGATCTC | To amplify in-*senRc* | |
| 1847for | GAGGAAGCCCGGTGCGAATCCGGCG | To amplify up-SCO1847 | |
| 1847rev | GGCACGCACGGCTCAACCGGCCGTG | To amplify up-SCO1847 | |
| 5226for | GGCACGTGCGCGCACGCCTGTCCAG | To amplify up-SCO5226 | |
| 5226rev | CTGGCGCGATGGTCACGTGGAGCAC | To amplify up-SCO5226 | |
| 4498for | GAAAGCATGGGTGTTAAAGTCGTGTG | To amplify up-SCO4498 | |
| 4498rev | CACACGGACATGCTCCGTAGGGGGAC | To amplify up-SCO4498 | |
| 6102for | CACGTCGACCTCGGCCGCATGTCC | To amplify up-SCO6102 | |
| 6102rev | GAGTGGCGGCCATGGTGTGACACG | To amplify up-SCO6102 | |
| 6102*for | CAAGCTTGAAGAGAGCACTCTCCAGC | To amplify up-SCO6102* | |
| 6102*rev | TTCAAGCTTGACAGATGGCGCTGG | To amplify up-SCO6102* | |
| 4229for | CTCGTACGTCCGTTTCGGGGGCTC | To amplify up-SCO4229 | |
| 4229rev | GTTCACGTCCATGTCTCCAGGTTAG | To amplify up-SCO4229 | |
| 4229*for | CAAGCTTGATCTTCGACGAGCCTAACCTGGAGAC | To amplify up-SCO4229* | |
| 4229*rev | TCGTCGAAGATCAAGCTTGAGCCATCCGGGTGG | To amplify up-SCO4229* | |
