# Supplementary material for: Deciphering the Transcriptional Response Mediated by the Redox-Sensing System HbpS-SenS-SenR from Streptomycetes
Source: PLoS One. 2016 Aug 19;11(8):e0159873. doi: 10.1371/journal.pone.0159873 (PMC4991794; doi:10.1371/journal.pone.0159873)
Supplement: S2 Table — (DOCX) [file pone.0159873.s004.docx]

**S2 Table. Summary of sequencing and mapping statistics**

|  | WT+^a^ | WT-^a^ | ∆*hsr*+^a^ | ∆*hsr*-^a^ |
| --- | --- | --- | --- | --- |
| Total reads | 2 x 5.16 | 2 x 3.11 | 2 x 4.50 | 2x 3.04 |
| Total mappings | 4.97 | 2.93 | 4.33 | 2.91 |
| Mapping of CPR^b^ | 4.89 | 2.89 | 4.26 | 2.87 |
| Mapping of single mapping reads | 0.08 | 0.05 | 0.07 | 0.05 |

^a^in million

^b^CPR: combined pair of reads
